# Does infant birthweight percentile identify mothers at risk of severe morbidity? A Canadian population-based cohort study

**DOI:** 10.1186/s40748-025-00217-8

**Published:** 2025-07-03

**Authors:** Joel G. Ray, Howard Berger, Kazuyoshi Aoyama, Jocelynn L. Cook, Kayvan Aflaki, Alison L. Park

**Affiliations:** 1 Departments of Medicine and Obstetrics and Gynaecology St. Michael’s Hospital, University of Toronto, 30 Bond Street, Toronto, ON M5B 1W8 Canada; 2 ICES, Toronto, ON Canada; 3 Department of Medicine, St. Michael’s Hospital, Toronto, ON Canada; 4 Department of Obstetrics and Gynaecology, St. Michael’s Hospital, Toronto, ON Canada; 5 Department of Anesthesia and Pain Medicine, The Hospital for Sick Children, Toronto, ON Canada; 6 Child Health Evaluative Sciences, The Hospital for Sick Children, Toronto, ON Canada; 7 Department of Obstetrics and Gynaecology, University of Ottawa, Ottawa, ON Canada; 8 The Society of Obstetricians and Gynaecologists of Canada, Ottawa, ON Canada; 9 Institute of Medical Science, University of Toronto, Toronto, ON Canada

**Keywords:** Severe maternal morbidity, Maternal mortality, Birthweight, Birthweight percentile, Fetal growth, Infant mortality, Neonatal mortality

## Abstract

**Background:**

There is a reverse J-shaped relation between newborn weight percentile and risk of perinatal mortality. Perinatal mortality itself is associated with severe maternal morbidity and mortality (SMM-M) around the index pregnancy, likely because the two share common etiologies, including placental dysfunction. We evaluated an infant’s birthweight percentile and risk of its mother experiencing SMM-M.

**Methods:**

This population-based cohort study was completed within a universal healthcare system in Ontario, Canada. Included were 2,203,490 singleton livebirths between 2002 and 2020. The study exposure was infant birthweight percentile for gestational age and sex. The 25th to 75th percentile served as the referent. The main outcome was SMM-M arising from 23 week’s gestation up to 42 days postpartum. Multivariable modified Poisson regression generated relative risks (aRRs) and 95% confidence intervals (CI), adjusted for maternal age, income, rurality, pre-existing diabetes and hypertension.

**Results:**

A J-shaped relation was seen between birthweight and risk of SMM-M. Relative to the 25th to 75th (15.0 per 1000 livebirths), the aRR of SMM-M was 1.27 (95% CI 1.21, 1.32) at 5th to < 10th, 1.40 (95% CI 1.28, 1.53) at 2nd to < 3rd, and 1.48 (95% CI 1.36, 1.62) at < 1st birthweight percentile. At higher birthweights, the aRR was 1.16 (95% CI 1.11, 1.21) at 90th to < 95th, 1.24 (95% CI 1.13, 1.36) at 95th to < 96th, and 1.73 (95% CI 1.60, 1.87) at > 99th percentile.

**Conclusion:**

There is a J-shaped relation between infant birthweight and risk of its mother experiencing SMM-M, likely due to shared risk factors and a common pathogenesis.

**Supplementary Information:**

The online version contains supplementary material available at 10.1186/s40748-025-00217-8.

## Abstract (social media quote)

**Social media quote:**

Study of 2.2 million births showed a J-shaped relation between newborn birthweight percentile and severe maternal morbidity.

**Synopsis:**

Study question: What is the association between newborn birthweight percentile and severe maternal morbidity or death (SMM-M)?

**What is already known:**

There exists a reverse J-shaped relation between newborn weight percentile and risk of perinatal mortality. Perinatal mortality itself is associated with severe maternal morbidity and mortality (SMM-M) around the index pregnancy, likely because the two share common etiologies, including placental dysfunction.

**What this study adds:**

A J-shaped relation was seen between infant birthweight percentile and risk of SMM-M. Identifying fetuses/newborns at extremes of small- and large-for-gestational age birthweight may provide a novel opportunity to detect and/or mitigate maternal morbidity.

**Supplementary Information:**

The online version contains supplementary material available at 10.1186/s40748-025-00217-8.

## Background

Severe maternal morbidity (SMM) -- a composite measure of acute morbidity -- is highly predictive of maternal hospital length of stay and maternal mortality. [1] SMM, which affects about 1.6% of pregnancies and largely arises around the time of the index delivery, [2–4] is also associated with infant mortality and stillbirth. [5] Some common pre-existing maternal chronic conditions, such as chronic hypertension and diabetes mellitus, as well as obesity and nulliparity, are established risk factors for SMM or maternal mortality. [6- 9] Some of these conditions are also predictors of both poor [10] and excessive [11, 12, 13] fetal growth. *Hence*,* SMM and abnormal fetal growth often share similar pre-existing maternal risk factors and a common pathogenesis*,* including placental dysfunction* (Figure S1).

There is a recognized reverse J-shaped relation between abnormal fetal growth and the risk of both stillbirth [12, 14, 15] and infant mortality, [16] especially at extremes of small (SGA) (e.g., < 1st percentile) and large (LGA) (e.g., > 99th percentile) for gestational age birthweight, as well as by the greater degree of prematurity. What remains unstudied, however, is the relation between extremes of abnormal birthweight percentile among liveborns and the risk of that newborn’s mother experiencing SMM or maternal mortality (SMM-M). In this regard, exclusively studying liveborn infants has some advantages over including stillbirths. Stillbirths are relatively uncommon, comprising about 6 in every 1000 births, [14, 17] and gestational age at birth and corresponding birthweight percentile are more accurately captured in a standardized manner for liveborn than stillborn fetuses, since an intrauterine fetal death may occur days in advance of its recognition or delivery.

It is implausible that abnormal fetal growth can cause SMM-M, per se. Rather, the current study was undertaken to explore the risk of a woman experiencing SMM-M in relation to her liveborn infant’s weight percentile to elucidate how extremes of SGA and LGA may share common pathogenic mechanisms with SMM-M, some of which may be modifiable upstream.

## Methods

### Study design and data sources

This population-based cohort study was completed using administrative data collected within Ontario’s universal health care system, as described elsewhere, [1, 5, 7] and listed in Table S1. These datasets are linked using unique encoded identifiers and analyzed at ICES, and have been well validated and studied in several prior studies. [1, 18, 19, 20].

The use of data in this project was authorized under Sect. 45 of Ontario’s Personal Health Information Protection Act, which does not require review by a Research Ethics Board.

### Population

We identified all singleton hospital livebirths born at ≥ 23 weeks’ gestation within the province of Ontario between September 1, 2002, and March 31, 2020. Excluded were non-Ontario resident mothers, women aged < 10 or > 55 years, stillbirths, multifetal births, and newborns with missing gestational age at birth, birthweight or sex (Table S1). Multifetal pregnancies were excluded, as they are more likely to experience poorer fetal growth [21, 22] and higher maternal morbidity. [23].

### Exposures

The main study exposure was an infant’s birthweight percentile for sex and gestational age, using a percentile curve for the general Ontario population. [24, 25, 26] Implausible birthweight for gestational age was handled according to the method by Alexander et al. [27].

### Outcomes

SMM was defined using a modified version that was developed and validated by the Canadian Perinatal Surveillance System, which is predictive of both maternal mortality and hospital length of stay [1], [4] (Table S1). SMM includes more than 40 unique indicators, based on International Classification of Diseases Canada (ICD-10-CA) diagnostic codes and Canadian Classification of Health Interventions (CCI) procedural codes, such as ICU admission, eclampsia and severe postpartum haemorrhage, but does not include isolated red cell transfusion, [1] since the latter tends to generate false-positive cases. [28] Leading SMM indicators from a similar Ontarian cohort and era are listed elsewhere (https://www.ncbi.nlm.nih.gov/pmc/articles/PMC6324398/table/zoi180201t2/).^1^ Maternal deaths occurring within or outside of a hospital setting were identified in the Registered Persons Database.

SMM-M was principally defined herein as that arising from 23 weeks’ gestation up to 6 weeks (i.e., 42 days) after the index delivery. As a secondary outcome, SMM-M was re-defined at any time between the index delivery hospitalization admission date and up to 6 weeks after the delivery. The latter approach was to tighten the time interval between newborn weight measurement and the diagnosis of SMM-M.

### Statistical analyses

Baseline newborn and maternal characteristics were presented as means, medians or proportions.

Univariable fractional polynomial regression and the RA2 selection algorithm [29, 30] optimally identified a curvilinear U-shaped-like relation between newborn weight percentile and SMM-M) from 23 weeks’ gestation up to 42 days postpartum, with a birthweight at the 25th to 75th percentile as the reference group (Figure S2). This reference group has been used elsewhere, [31] and corresponds to a category in which SMM-M remains below the overall rate of 16 per 1000 births previously described in Canada [32, 33] (Figure S2).

The *Main model* estimated the relative risk (RR) and 95% confidence interval (CI) of the association between infant birthweight percentile and SMM-M using modified Poisson regression with a robust error variance. Generalized estimating equations with an exchangeable correlation structure accounted for correlated errors in the case of more than one birth event within the same woman. [34, 35] At under the 25th percentile, categories of birthweight percentile were then presented by fifths down to the 5th percentile, and then by single percentiles, since the greatest risk of SMM-M was seen at extremes of SGA (Figure S2). Likewise, above the 75th percentile, increments of fifths were used up to the 95th percentile and then by single percentiles beyond. RRs were adjusted for maternal age (continuous), residential income quintile (Q1 or missing, Q2, Q3, Q4, vs. Q5) and rural residence– each at the time of the index birth -- as well as diabetes mellitus and chronic hypertension within 2 years before the index birth (Table S1). All covariates were chosen a priori, based on our conceptual framework (Figure S1) and the near-complete availability of those important variables.

A re-analysis of the *Main model* replaced the general population birthweight curve with ones more specific to maternal ethnicity [24, 25, 26] (*Additional analysis A*).

The *Main model* of birthweight percentile and SMM-M was further stratified by important variables related to fetal growth and SMM-M, including pre-existing type 1 or 2 diabetes mellitus, chronic hypertension, maternal parity (parous or nulliparous), and mode of birth (vaginal or Caesarean) (Figure S1). As BMI was often not collected, a complete case analysis was undertaken, stratifying by a BMI < 30 or ≥ 30 kg/m^2^.

*Additional analysis B* used the same approach as in the *Main model* but partitioned the SMM outcome indicators, a priori, into those that might be more likely to be related to poor fetal growth -- especially mediated by placental dysfunction [36] (Figure S1) -- and those potentially less related to poor fetal growth (Table S2). In that modified model, maternal mortality was not included in the outcome.

All analyses were performed using SAS statistical software, version 9.4 (SAS Institute Inc., Cary, NC) and Excel for Macintosh, version 15.3.9 (Microsoft Corporation, Redmond, Washington).

### Missing data

In the case of missingness on a covariate with counts < 6, missingness was assigned to the referent category. No data imputation was otherwise indicated nor performed. As specified above, since BMI was often missing, a complete case analysis was undertaken, then stratifying by a BMI < 30 or ≥ 30 kg/m^2^.

### Ethics approval

The use of data in this project was authorized under Sect. 45 of Ontario’s Personal Health Information Protection Act, which does not require review by a Research Ethics Board.

## Results

**Fig. 1:**
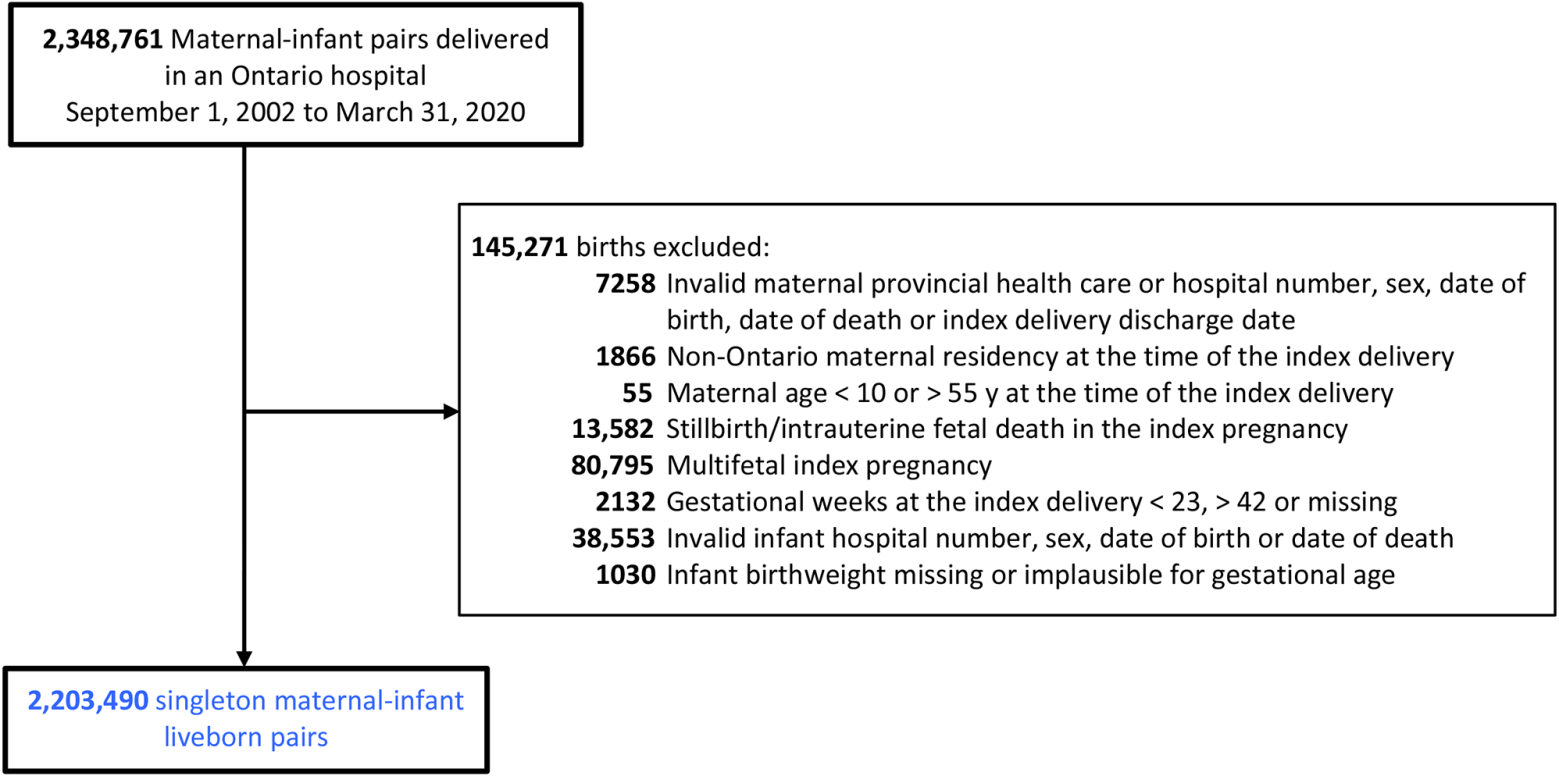
Flow diagram of cohort creation

Out of 2,348,761 maternal-infant liveborn pairs identified during the study period, 145,271 (6.2%) were excluded (Fig. 1). Among the 2,203,490 included maternal-infant liveborn pairs, 14.9% of women had pre-pregnancy diabetes, 15.9% chronic hypertension, 6.1% had a preterm birth in the index (i.e., same) pregnancy, 27.7% of births were by Caesarean section, and 4.3% of their infants had a congenital or chromosomal anomaly diagnosed in the first year of life (Table 1).

**Table 1:** Baseline characteristics of 2,203,490 Singleton liveborn infants and their mothers. All data are shown as a number (%) unless otherwise indicated

Characteristic	Value
Maternal age groups, y	
10–19	64,953 (2.9)
20–34	1,653,579 (75.0)
35–39	400,052 (18.2)
40–44	80,520 (3.7)
45–55	4386 (0.2)
Maternal World region of birth	
Canada or long-term resident of Canada	1,612,192 (73.2)
Caribbean or Sub-Saharan Africa	75,089 (3.4)
East Asia or Pacific	139,348 (6.3)
Hispanic America	44,179 (2.0)
Middle East or North Africa	64,432 (2.9)
South Asia	178,000 (8.1)
Western Nations or Europe	90,135 (4.1)
Unknown	115 (0.0)
Income quintile (Q) at the index delivery	
Q1 (lowest) or unknown	499,000 (22.6)
Q2	439,782 (20.0)
Q3	450,845 (20.5)
Q4	453,474 (20.6)
Q5 (highest)	360,389 (16.4)
Urban residence at index delivery	1,981,308 (89.9)
Parity	
0	972,647 (44.1)
1	798,266 (36.2)
2+	432,577 (19.6)
Body mass index category, kg/m^2 a^	
< 30	246,459 (11.1)
≥ 30	61,564 (2.8)
Unknown	1,895,467 (86.0)
Pre-pregnancy diabetes mellitus diagnosed within 2 y before, or at the time of, the index birth	
Absent Present	1,875,591 (85.1) 327,899 (14.9)
Chronic hypertension diagnosed within 2 y before, or at the time of, the index birth	
Absent Present	1,852,866 (84.1) 350,624 (15.9)
Mean (SD) gestational age at index birth, weeks	38.9 (1.8)
Preterm birth < 37 weeks’ gestation at the index delivery	
Absent Present	2,068,492 (93.4) 134,998 (6.1)
Caesarean birth at the index delivery	
Absent Present	1,593,171 (72.3) 610,319 (27.7)
Female newborn	
Absent Present	1,130,889 (51.3) 1,072,601 (48.7)
Congenital or chromosomal anomaly diagnosed in the first year of the infant’s life	
Absent Present	2,107,772 (95.7) 95,718 (4.3)

^a^Limited to 308,023 births (14.0% of all births) with known maternal pre-pregnancy body mass index

SD standard deviation; IQR interquartile range

There were 36,657 SMM-M outcome events (1.7 per 1000 births)– 1728 (4.7%) arising before the index birth and 34,929 (95.2%) during the index delivery hospitalization and up to 6 weeks thereafter.

**Fig. 2:**
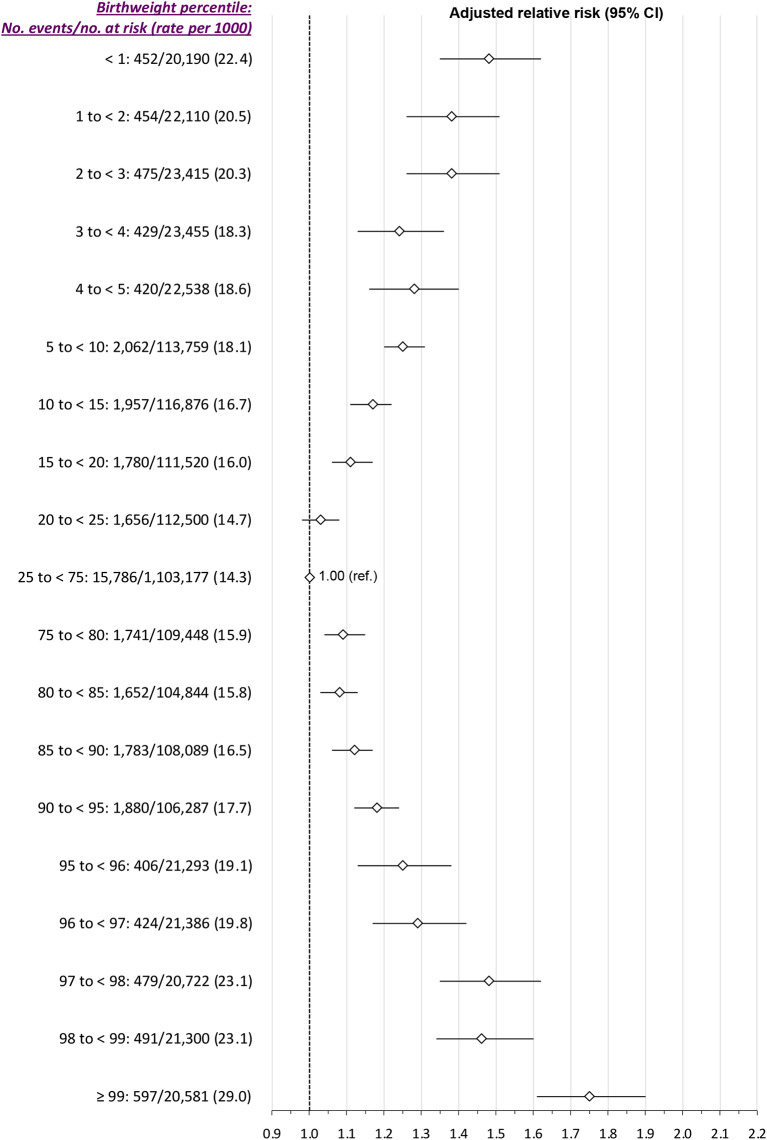
(Main model). Risk of severe maternal morbidity or death from 23 weeks’ gestation up to 6 weeks postpartum, in relation to newborn weight percentile. Relative risks (RR) are adjusted for maternal age, income quintile and rural residence– each at the time of the index birth -- as well as diabetes mellitus and chronic hypertension within 2 years before the index birth

**Fig. 3:**
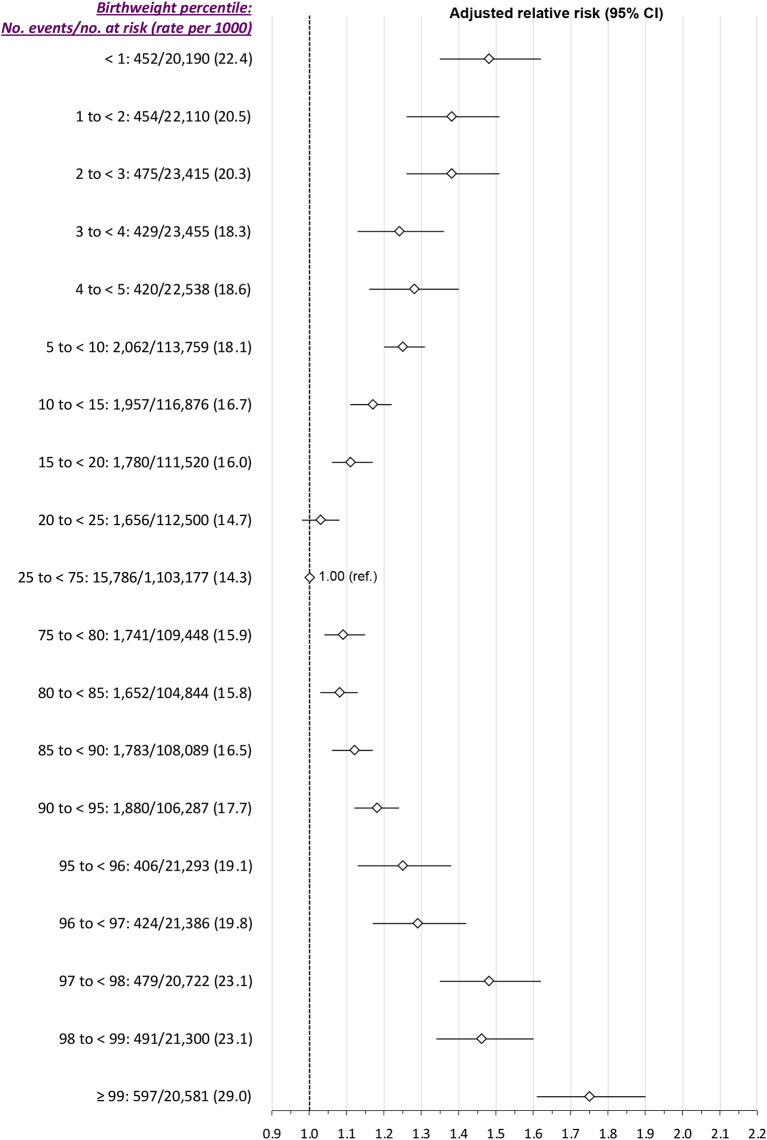
Risk of severe maternal morbidity or death from the index birth hospitalization up to up to 6 weeks postpartum, in relation to newborn weight percentile. Relative risks are adjusted for maternal age, income quintile and rural residence– each at the time of the index birth -- as well as diabetes mellitus and chronic hypertension within 2 years before the index birth

There was a J-shaped relation between birthweight percentile and the risk of SMM-M between 23 weeks’ gestation and 6 weeks postpartum (*Main model*, Fig. 2). At the higher birthweight percentiles, adjusting for study covariates attenuated the RRs in a more pronounced manner than at the lower percentiles. Relative to the 25th to 75th percentile birthweight (15.0 per 1000 livebirths), the aRR of SMM-M was 1.48 (95% CI 1.36, 1.62) at < 1st percentile, and 1.73 (95% CI 1.60, 1.87) at > 99th percentile (Fig. 2). Using birthweight curves specific to maternal ethnicity generated similar effect sizes and a similar J-shaped pattern (*Additional analysis A*, Figure S3). For the secondary outcome of SMM-M arising between the index delivery hospitalization and up to 6 weeks thereafter, a J-shaped pattern persisted (Fig. 3).

**Fig. 4:**
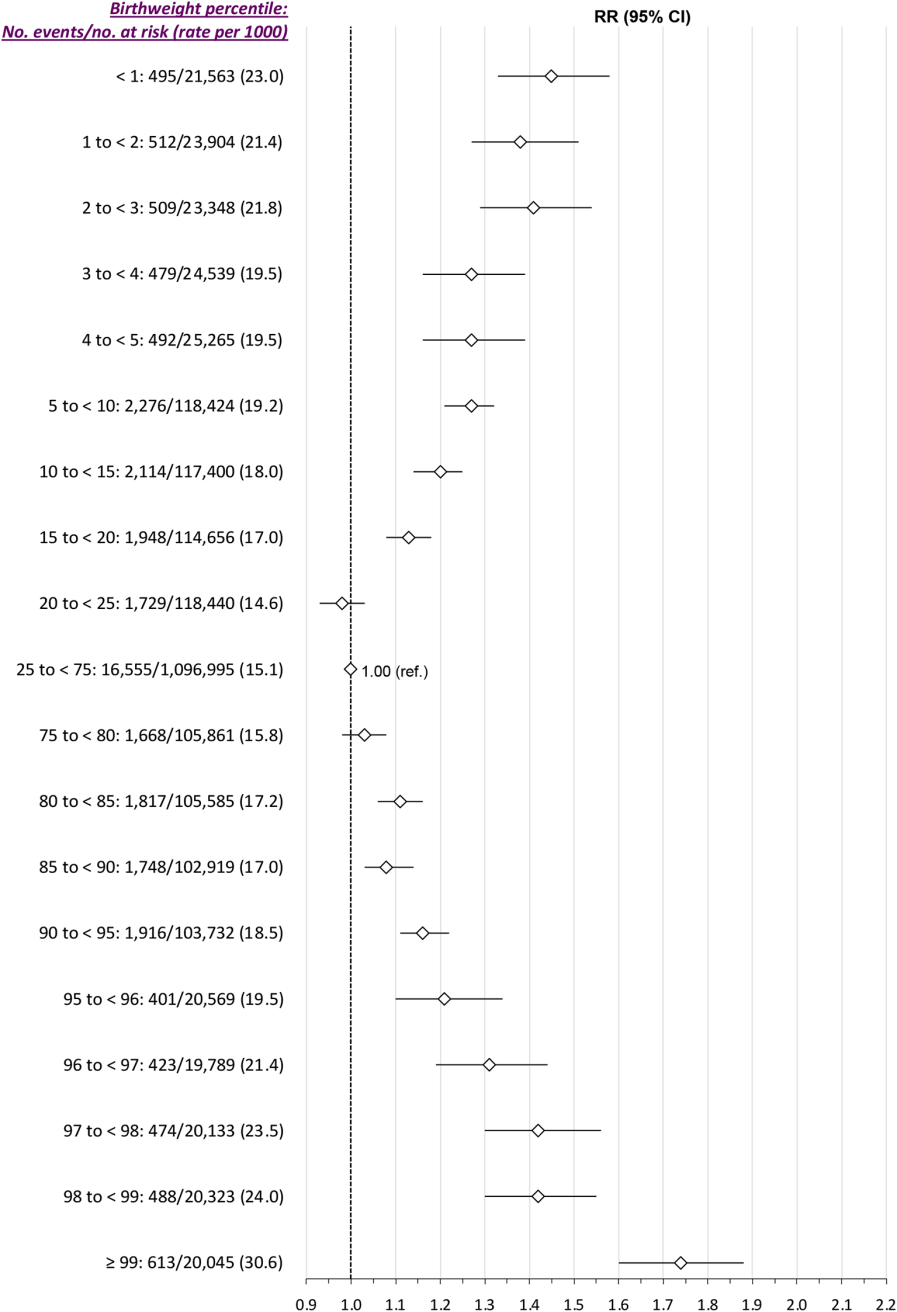
Risk of severe maternal morbidity or death from 23 weeks’ gestation up to up to 6 weeks postpartum, in relation to newborn weight percentile, stratified by the absence or presence pre-pregnancy diabetes mellitus. Relative risks (RR) are adjusted for maternal age, income quintile and rural residence– each at the time of the index birth -- as well as chronic hypertension within 2 years before the index birth

**Fig. 5:**
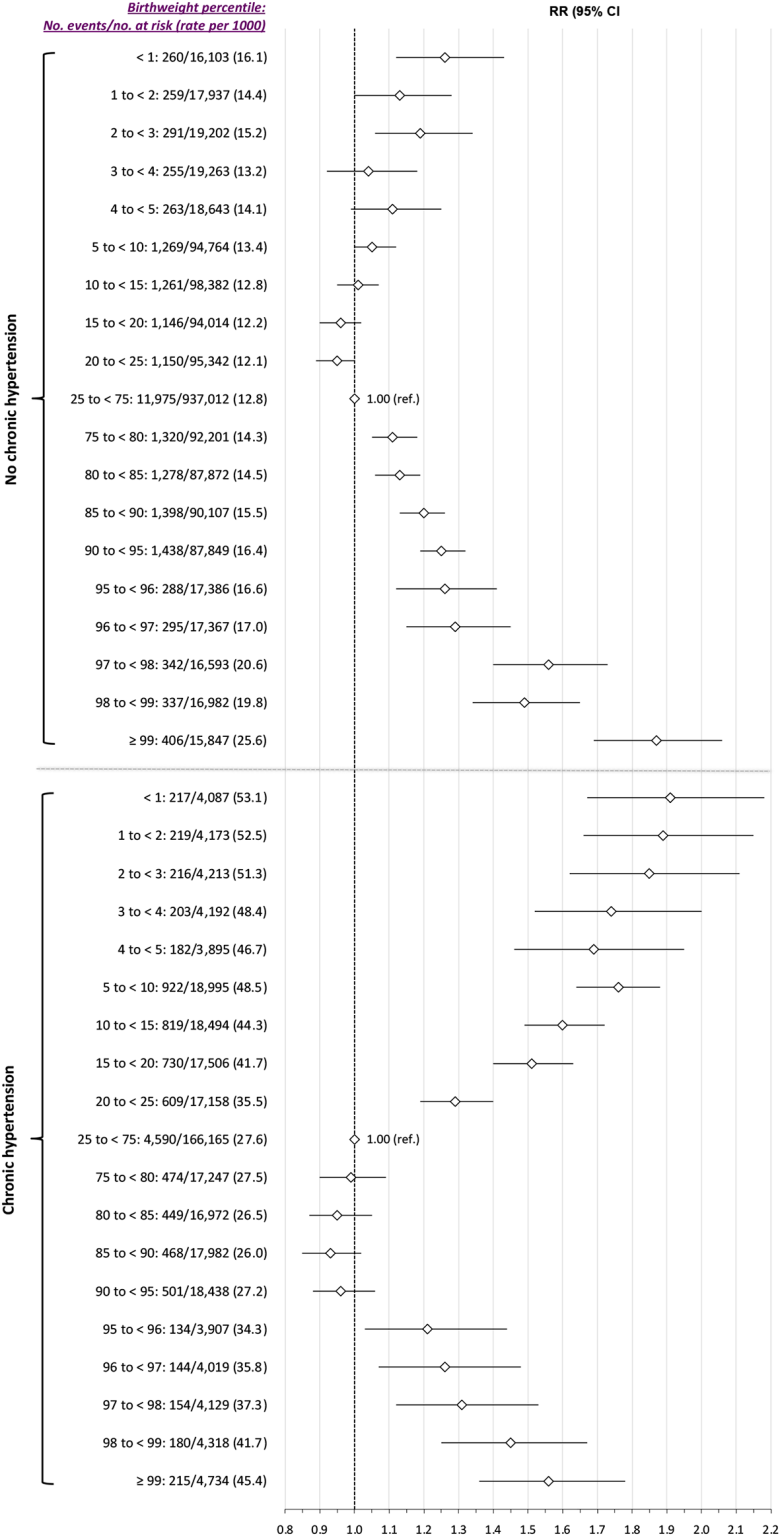
Risk of severe maternal morbidity or death from 23 weeks’ gestation up to up to 6 weeks postpartum, in relation to newborn weight percentile, stratified by the absence or presence of chronic hypertension. Relative risks (RR) are adjusted for maternal age, income quintile and rural residence– each at the time of the index birth -- as well as diabetes mellitus within 2 years before the index birth

In the *Main model*, the J-shaped pattern of birthweight percentile and SMM-M between 23 weeks’ gestation and 6 weeks postpartum remained in those with pre-existing diabetes, with a more U-shaped relation in women without diabetes (Fig. 4). SMM-M was also notably more prevalent among those with vs. without diabetes. For example, at the 97th to < 98th percentile birthweight, the rates were 32.0 and 21.5 per 1000 births, respectively. In women with chronic hypertension, a reverse J-shaped pattern was much more apparent (Fig. 5). Moreover, at almost all birthweight percentiles, SMM-M was more than twice as likely among those with than without chronic hypertension.

Stratification by pre-pregnancy BMI < 30 or ≥ 30 kg/m^2^ showed an attenuated U-shaped relation among the 308,023 pregnancies in whom BMI was recorded (Figure S4). Among parous women, a U-shaped pattern was seen (Figure S5, upper), but among nulliparous women, a pronounced J-shape pattern was noted, with markedly high rates of SMM-M at the upper birthweight percentiles (Figure S5, lower). The same respective patterns were seen for vaginal (Figure S6, upper) and Caesarean (Figure S6, lower) delivery.

Finally, upon partitioning the SMM outcome indicators into those assumed to be most likely related to poor fetal growth, such as severe preeclampsia or placental abruption (see Table S2), the reverse J-shaped relation was accentuated at the lowest birthweights (*Additional analysis B*, Figure S7, upper)– an effect markedly blunted for SMM outcome indicators considered less likely related to poor fetal growth (Figure S7, lower).

## Discussion

### Principal findings

In this novel large population-based study of 2.2 million singleton newborn-maternal pairs over an 18-year period, infant birthweight percentile showed an overall J-shaped relation to SMM-M arising in pregnancy or postpartum. Somewhat similar patterns varied according to whether the mother had pre-pregnancy diabetes or hypertension, parity, and gestational age and mode of birth.

### Strengths of the study

This well-powered study illuminated a curvilinear higher risk of SMM-M at more discrete birthweight percentiles, especially at < 5th and > 95th centiles. Furthermore, when we shortened the timeline between birthweight measurement and assessment of SMM-M around the time of birth, the J-shaped pattern remained strong, increasing the likelihood of a true relation between the two.

Except for home births, this study included all singleton hospital births within a universal health care system, thereby minimizing selection bias. As only singletons were studied, the relation between newborn weight and SMM-M might differ for multiples. [23] Despite their high specificity, ICD-10-CA hospitalization codes may lack sensitivity for identifying SMM, [28] likely leading to non-differential misclassification of the outcome and a bias towards the null. Conversely, despite the apparent use of appropriate codes, the rates of pre-pregnancy diabetes mellitus and chronic hypertension herein were much higher than expected. [37] One explanation is that these diagnostic codes were incorrectly applied to women with gestational diabetes mellitus and pregnancy-induced hypertension, respectively. In the presence and absence of these pre-existing conditions, the relation between newborn weight percentile and SMM persisted, In neither case is this potential over-classification of chronic hypertension or pre-pregnancy diabetes mellitus “hidden” from the reader. Rather, as shown in Figs. 4 and 5, in the presence or absence of these pre-existing conditions, the relation between newborn weight percentile and SMM persisted.

Recorded birthweight, gestational age and newborn sex are the determinants of birthweight percentile. In Ontario, at least 95% of pregnancies have an antenatal ultrasound, [38] and the currently used administrative datasets have a high accuracy for determining gestational age at birth. [39] The birthweight percentiles used herein were previously derived using the Ontario population of liveborn singletons, and then further considered maternal World region of birth as a proxy for maternal ethnicity. [24, 25, 26] The current Canadian definition of SMM has been validated against maternal mortality and maternal hospital length of stay and is like that used in the U.S. and by the WHO. [1, 3, 4, 26] Other women who experienced SMM and then had a stillbirth, or had not given birth at all (as in the case of a rare maternal death whilst pregnant), were not captured herein. Even so, stillbirths are relatively uncommon, [14, 17] stillbirth birthweight measurement may be less accurate, and 95% of SMM-M events herein arose between the index birth hospitalization and the 6-week postpartum period.

### Limitations of the data

While the current study considered several common factors associated with abnormal fetal growth, [36] other unmeasured confounders could explain some of our findings, such as prior pregnancy morbidity, acquired chorioamnionitis or other serious infections, net pregnancy weight gain and smoking (Figure S1). Nevertheless, in Ontario, the prevalence of smoking in pregnancy is only 8.8% and heavily correlated with income status and rurality. [40] Importantly, among women with diabetes and hypertension, we did not account for glycemic or blood pressure control, respectively, nutrition, or related medication use. Thus, residual confounding could explain the study findings. For the observed aRR of 1.48 for SMM-M at a birthweight < 1st percentile, and aRR of 1.73 at > 99th percentile, for example, the calculated E-values would be 2.32 and 2.85, respectively. Those E-values suggest that unmeasured confounding could explain away those associations only if those unmeasured confounders more than doubled the risk of SMM among either the unexposed or the exposed. [41].

Maternal BMI was often not known in this study. A low and a high BMI may distinctly confound the relation between fetal growth and SMM-M (Figure S1). In a study completed in Ontario using similar datasets, we contrasted those with a recorded vs. a missing BMI and found little difference in demographic or clinical variables between the two groups. [42] Certainly, a more robust exploration of the interaction between BMI and birthweight percentiles can be considered in a future study. Also needed is an evaluation of a sub-set of SMM indicators expected to be more related to excess fetal growth, akin to Additional analysis B (Table S2, Figure S7).

### Interpretation

Prior studies are lacking on birthweight percentiles and SMM-M risk. One insightful study disaggregated its composite of *neonatal* morbidity (CNM) into that potentially related to hypoxic newborn events (e.g., bronchopulmonary dysplasia or hypoxic-ischemic encephalopathy) and that related to traumatic newborn events (e.g., osseous fracture or brachial plexus palsy). [43] Therein, among newborns SGA < 10th percentile, hypoxic CNM was 1.44 times higher compared to the 10th to 90th percentile birthweight referent, while traumatic CNM was 1.88 times higher at > 90th percentile birthweight. [43] In analogy to the CNM example, there was a heightened relation between newborn SGA and certain SMM indicators posited to be more likely related to poor fetal growth (Table S2), as shown in Figure S7. This type of approach also underscores the value of performing additional analyses that can more specifically align certain SMM indicators with the risk factor being tested. [44] It is well recognized that placental vascular disease can restrict fetal growth and predispose to preterm birth, [45] while also heightening the risk of severe preeclampsia, placental abruption and antepartum haemorrhage (Figure S1), and the ensuing need for maternal critical care. [45].

In the presence of chronic hypertension, severe SGA had a pronounced reverse J-shaped relation to SMM-M (Fig. 5, lower). Women with hypertension have the highest rate of placental abruption, especially at the lowest birthweight centiles. [46] Severe LGA was associated with SMM-M herein. This pattern was seen in the *Main model* and was more pronounced, both in absolute and relative terms, in women with pre-pregnancy diabetes (Fig. 4). Others have described a higher risk of severe postpartum haemorrhage and advanced perineal trauma in women giving birth to an infant weighing > 4000 g, especially > 4500 g. [47].

At least one third of cases of SMM are believed to be preventable, [48] and about 95% of SMM-M events occurred herein at the index delivery or soon thereafter. The latter strongly suggests that abnormal fetal growth develops gradually, antecedent to SMM-M; nevertheless, they are each often related to placental vascular disease, as discussed above.

In women with pre-pregnancy diabetes, observational data suggest that improved peri-conceptional glycemic control is associated with a reduction in SMM-M, preterm birth and perinatal mortality. [49] Multiple randomized clinical trials (RCT) have also shown a reduced risk of fetal growth restriction, preterm birth and severe preeclampsia with the initiation of low-dose aspirin before 16 weeks’ gestation in high-risk women. [50] RCTs also suggest that blood pressure lowering therapy can prevent severe hypertension, severe preeclampsia, placental abruption and preterm birth but may heighten the risk of SGA. [51].

An intrapartum Maternal Early Warning Trigger tool -- based on maternal vital signs around the time of labour and delivery -- may reduce the incidence of SMM. [52] Whether the addition of a relatively static measure, like fetal or newborn weight percentile, improves that tool is worthy of exploration, while considering those SMM indicators likely to be paired with severe SGA or LGA. Furthermore, there exists the possibility that SMM-M might be forecasted by upstream indicators of abnormal fetal growth. Yet, published clinical prediction models for SMM-M have not included fetal growth measures acquired by prenatal ultrasonography. [53, 54] Furthermore, among low-risk pregnancies, the additional measurement of a cerebroplacental ratio near to term was recently shown to reduce severe neonatal morbidity compared with fetal growth assessment alone. [55] While high-quality studies of fetal growth velocity [56] and Doppler-based measurement of fetal well-being [55] have assessed neonatal outcomes, maternal adversity was not evaluated therein. Hence, the added value of such information, often available from within existing electronic prenatal records, should be tested within a related clinical prediction model of SMM-M. Finally, in the prediction of SMM-M, artificial intelligence-based deep learning models might not only utilize text-based measures of fetal growth, but also real-time sonographic images. [57] Such analyses may reveal other patterns describing fetal growth and ensuing SMM-M that are not necessarily like the curvilinear relations seen herein.

## Conclusions

A J-shaped relation exists between discrete birthweight percentiles and the risk of SMM-M. It should be determined whether identifying fetuses at extremes of SGA and LGA can help detect and mitigate impending maternal illness in some cases.

## Electronic supplementary material

Below is the link to the electronic supplementary material.

Supplementary Material 1

## Data Availability

No datasets were generated or analysed during the current study.

## References

[1] Ray JG, Park AL, Dzakpasu S, et al. Prevalence of severe maternal morbidity and factors associated with maternal mortality in Ontario, Canada. JAMA Netw Open. 2018;1:e184571. 30646359 10.1001/jamanetworkopen.2018.4571 PMC6324398

[2] Callaghan W, Creanga AA, Kuklina EV. Severe maternal morbidity among delivery and postpartum hospitalizations in the united States. Obstet Gynecol. 2012;120:1029–36. 23090519 10.1097/aog.0b013e31826d60c5

[3] Aoyama K, Ray JG, Pinto R, et al. Temporal variations in incidence and outcomes of critical illness among pregnant and postpartum women in Canada: a population-based observational study. J Obstet Gynaecol Can. 2019;41:631–40. 30385209 10.1016/j.jogc.2018.07.021

[4] Dzakpasu S, Deb-Rinker P, Arbour L, Darling EK, Kramer MS, Liu S, Luo W, Murphy PA, Nelson C, Ray JG, Scott H, VandenHof M, Joseph KS. Severe maternal morbidity surveillance: monitoring pregnant women at high risk for prolonged hospitalisation and death. Paediatr Perinat Epidemiol. 2020;34:427–39. 10.1111/ppe.12574. 31407359 10.1111/ppe.12574 PMC7383693

[5] Aoyama K, Park AL, Davidson AJF, et al. Severe maternal morbidity and infant mortality in Canada. Pediatrics. 2020;146:e20193870. 32817396 10.1542/peds.2019-3870

[6] Hitti J, Sienas L, Walker S, et al. Contribution of hypertension to severe maternal morbidity. Am J Obstet Gynecol. 2018;219:e4051–7. 10.1016/j.ajog.2018.07.002 30012335

[7] Davidson AJF, Park AL, Berger H, et al. Risk of severe maternal morbidity or death in relation to elevated hemoglobin A1c preconception, and inn early pregnancy: a population-based cohort study. PLoS Med. 2020;17:e1003104. 32427997 10.1371/journal.pmed.1003104 PMC7236974

[8] Lisonkova S, Muraca GM, Potts J, et al. Association between prepregnancy body mass index and severe maternal morbidity. JAMA. 2017;18:1777–86. 10.1001/jama.2017.16191 PMC5820710 29136442

[9] Gray KE, Wallace ER, Nelson KR, et al. Population-based study of risk factors for severe maternal morbidity. Pediatr Perinal Epidemiol. 2012;26:506–14. 10.1111/ppe.12011 PMC3498497 23061686

[10] Ankumah NE, Sibai BM. Chronic hypertension in pregnancy: diagnosis, management, and outcomes. Clin Obstet Gynecol. 2017;60:206–14. 28005588 10.1097/GRF.0000000000000255

[11] Ray JG, Vermeulen MJ, Shapiro JL, et al. Maternal and neonatal outcomes in pregestational and gestational diabetes mellitus, and the influence of maternal obesity and weight gain: the DEPOSIT study. Diabetes endocrine pregnancy outcome study in Toronto. QJM. 2001;94:347–56. 11435630 10.1093/qjmed/94.7.347

[12] Page JM, Allshouse AA, Cassimatis I, et al. Characteristics of stillbirths associated with diabetes in a diverse U.S. Cohort. Obstet Gynecol. 2020;136:1095–102. 33156199 10.1097/AOG.0000000000004117 PMC7680368

[13] Santos S, Voerman E, Amiano P, et al. Impact of maternal body mass index and gestational weight gain on pregnancy complications: an individual participant data meta-analysis of European, North American and Australian cohorts. BJOG - Int J Obstet Gyn. 2019;126:984–95. 10.1111/1471-0528.15661 PMC6554069 30786138

[14] Ray JG, Urquia ML. Risk of stillbirth at extremes of birth weight between 20 to 41 weeks gestation. J Perinatol. 2012;32:829–36. 22595964 10.1038/jp.2012.60

[15] Moraitis AA, Wood AM, Fleming M, et al. Birth weight percentile and the risk of term perinatal death. Obstet Gynecol. 2014;124:274–83. 25004344 10.1097/AOG.0000000000000388

[16] Ludvigsson JF, Lu D, Hammarström L, et al. Small for gestational age and risk of childhood mortality: A Swedish population study. PLoS Med. 2018;15:e1002717. 30562348 10.1371/journal.pmed.1002717 PMC6298647

[17] Fenady V, Koopmans L, Middleton P, et al. Major risk factors for stillbirth in high-income countries: a systematic review and meta-analysis. Lancet. 2011;377:1331–40. 21496916 10.1016/S0140-6736(10)62233-7

[18] Joseph KS, Fahey J, Canadian Perinatal Surveillance System. Validation of the perinatal data in the discharge abstract database of the Canadian Institute for health information. Chronic Dis Can. 2009;29:96–100. 19527567

[19] Ray JG, Urquia ML, Berger H, et al. Maternal and neonatal separation and mortality associated with concurrent admissions to intensive care units. Can Med Assoc J. 2012;184:E956–62. 23091180 10.1503/cmaj.121283 PMC3519169

[20] Ray JG, Park AL, Fell DB. Mortality in infants affected by preterm birth and severe small-for-gestational age birth weight. Pediatrics. 2017;140:e20171881. 29117948 10.1542/peds.2017-1881

[21] Ananth CV, Vintzileos AM, Shen-Schwarz S, et al. Standards of birth weight in twin gestations stratified by placental chorionicity. Obstet Gynecol. 1998;91:917–24. 9610996 10.1016/s0029-7844(98)00052-0

[22] Smith LK, Manktelow BN, Draper ES, et al. Trends in the incidence and mortality of multiple births by socioeconomic deprivation and maternal age in England: population-based cohort study. BMJ Open. 2014;4:e004514. 24699461 10.1136/bmjopen-2013-004514 PMC3987713

[23] Black CM, Vesco KK, Mehta V et al. Incidence of severe maternal morbidity during delivery hospitalization in US commercially insured and medicaid populations. J Womens Health 2021. Epub ahead of print. 10.1089/jwh.2020.8556 PMC11896018 33891488

[24] Ray JG, Sgro M, Mamdani MM, et al. Birth weight curves tailored to maternal world region. J Obstet Gynaecol Can. 2012;34:159–71. 22340065 10.1016/S1701-2163(16)35159-3

[25] Urquia ML, Berger H, Ray JG, et al. Risk of adverse outcomes among infants of immigrant women according to birth-weight curves tailored to maternal world region of origin. Can Med Assoc J. 2015;187:E32–40. 25384653 10.1503/cmaj.140748 PMC4284190

[26] Park AL, Tu K, Ray JG, Canadian Curves Consortium. Differences in growth of Canadian children compared to the WHO 2006 child growth standards. Paediatr Perinat Epidemiol. 2017;31:452–62. 10.1111/ppe.12377. 28692179 10.1111/ppe.12377

[27] Alexander GR, Himes JH, Kaufman RB, Mor J, Kogan M. A United States national reference for fetal growth. Obstet Gynecol. 1996;87(2):163-8. 10.1016/0029-7844(95)00386-X. PMID: 8559516. 10.1016/0029-7844(95)00386-X 8559516

[28] Snowden JM, Lyndon A, Kan P, El Ayadi A, Main E, Carmichael SL. Severe maternal morbidity: A comparison of definitions and data sources. Am J Epidemiol. 2021;190:1890–7. 10.1093/aje/kwab077. 33755046 10.1093/aje/kwab077 PMC8579027

[29] Royston P, Altman DG. Regression using fractional polynomials of continuous covariates: parsimonious parametric modeling. Appl Stat - J Roy St C. 1994;43:429–67.

[30] Ambler G, Royston P. Fractional polynomial model selection procedures: investigation of type I error rate. J Stat Comput Simul. 2001;69:89–108.

[31] Xu H, Simonet F, Luo ZC. Optimal birth weight percentile cut-offs in defining small- or large-for-gestational-age. Acta Paediatr. 2010;99:550–55. 20064130 10.1111/j.1651-2227.2009.01674.x

[32] Dzakpasu S, Deb-Rinker P, Arbour L, et al. Severe maternal morbidity surveillance: monitoring pregnant women at high risk for prolonged hospitalisation and death. Paediatr Perinat Epidemiol. 2020;34:427–39. 31407359 10.1111/ppe.12574 PMC7383693

[33] Aoyama K, Pinto R, Ray JG, et al. Association of maternal age with severe maternal morbidity and mortality in Canada. JAMA Netw Open. 2019;2:e199875. 31441937 10.1001/jamanetworkopen.2019.9875 PMC6714030

[34] Yelland LN, Salter AB, Ryan P. Performance of the modified Poisson regression approach for estimating relative risks from clustered prospective data. Am J Epidemiol. 2011;174:984–92. 21841157 10.1093/aje/kwr183

[35] Hubbard AE, Ahern J, Fleischer NL, et al. To GEE or not to GEE: comparing population average and mixed models for estimating the associations between neighborhood risk factors and health. Epidemiology. 2010;21:467–74. 20220526 10.1097/EDE.0b013e3181caeb90

[36] Ananth CV. Ischemic placental disease: a unifying concept for preeclampsia, intrauterine growth restriction, and placental abruption. Semin Perinatol. 2014;38:131–2. 24836823 10.1053/j.semperi.2014.03.001

[37] Dzakpasu S, Nelson C, Darling EK, Edwards W, Murphy PA, Scott H, Van den Hof M, Ray JG, Canadian Perinatal Surveillance System. Trends in rate of hypertensive disorders of pregnancy and associated morbidities in Canada: a population-based study (2012–2021). CMAJ. 2024;196(E897–E904). 10.1503/cmaj.231547. 10.1503/cmaj.231547 PMC11286177 39074863

[38] You JJ, Alter DA, Stukel TA, et al. Proliferation of prenatal ultrasonography. Can Med Assoc J. 2015;187:143–51. 10.1503/cmaj.090979 PMC2817321 20048009

[39] Flitzpatrick T, Wilton AS, Guttmann A. Development and validation of a simple algorithm to estimate common gestational age categories using standard administrative birth record data in Ontario, Canada. J Obstet Gynaecol. 2021;41:207–11. 32590915 10.1080/01443615.2020.1726304

[40] Al-Sahab B, Saqib M, Hauser G, Tamim H. Prevalence of smoking during pregnancy and associated risk factors among Canadian women: a National survey. BMC Pregnancy Childbirth. 2010;10:24. 10.1186/1471-2393-10-24. 20497553 10.1186/1471-2393-10-24 PMC2885995

[41] Mathur MB, Ding P, Riddell CA, VanderWeele TJ. Web site and R package for computing E-values. Epidemiology. 2018;29(5):e45–7. 29912013 10.1097/EDE.0000000000000864 PMC6066405

[42] Cohen E, Fu L, Brown HK, Grandi SM, Boblitz A, Fang J, Austin PC, Nathwani AA, Szentkúti P, Horváth-Puhó E, Sørensen HT, Ray JG. Adverse perinatal events and maternal interpregnancy weight change: A population-based observational study. Int J Gynaecol Obstet. 2024;165:792–800. 10.1002/ijgo.15296. 38100266 10.1002/ijgo.15296

[43] Chauhan SP, Rice MM, Grobman WA, et al. Neonatal morbidity of Small- and Large-for-Gestational-Age neonates born at term in uncomplicated pregnancies. Obstet Gynecol. 2017;130:511–9. 28796674 10.1097/AOG.0000000000002199 PMC5578445

[44] Davidson JF, Park AL, Ray JG. Navigating severe maternal morbidity using big data: green, yellow, and red flags for researchers. Obstet Med. 2019;12:105–6. 31523265 10.1177/1753495X19872880 PMC6734634

[45] Villar J, Carroli G, Wojdyla D, et al. Preeclampsia, gestational hypertension and intrauterine growth restriction, related or independent conditions? Am J Obstet Gynecol. 2006;194:921–31. 16580277 10.1016/j.ajog.2005.10.813

[46] Ananth CV, Peltier MR, Kinzler WL, et al. Chronic hypertension and risk of placental abruption: is the association modified by ischemic placental disease? Am J Obstet Gynecol. 2007;197:e2731–7. 10.1016/j.ajog.2007.05.047 17826417

[47] Beta J, Khan N, Fiolna M, et al. Maternal and neonatal complications of fetal macrosomia: cohort study. Ultrasound Obst Gyn. 2019;54:319–25. 10.1002/uog.20278 30938000

[48] Lawton BA, MacDonald EJ, Stanley J, et al. Preventability review of severe maternal morbidity. Acta Obstet Gynecol Scand. 2019;98:515–22. 30586147 10.1111/aogs.13526

[49] Davidson AJF, Park AL, Berger H, Aoyama K, Harel Z, Cohen E, Cook JL, Ray JG. Association of improved periconception hemoglobin A1c with pregnancy outcomes in women with diabetes. JAMA Netw Open. 2020;3:e2030207. 33355674 10.1001/jamanetworkopen.2020.30207 PMC7758806

[50] Roberge S, Nicolaides K, Demers S, Hyett J, Chaillet N, Bujold E. The role of aspirin dose on the prevention of preeclampsia and fetal growth restriction: systematic review and meta-analysis. Am J Obstet Gynecol. 2017;216:110–e1206. 10.1016/j.ajog.2016.09.076. 27640943 10.1016/j.ajog.2016.09.076

[51] Abe M, Arima H, Yoshida Y, Fukami A, Sakima A, Metoki H, Tada K, Mito A, Morimoto S, Shibata H, Mukoyama M. Optimal blood pressure target to prevent severe hypertension in pregnancy: A systematic review and meta-analysis. Hypertens Res. 2022;48. 10.1038/s41440-022-00853-z.48. 10.1038/s41440-022-00853-z 35136186

[52] Edwards W, Dore S, van Schalkwyk J, Armson BA. Prioritizing maternal sepsis: National adoption of an obstetric early warning system to prevent morbidity and mortality. J Obstet Gynaecol Can. 2020;42:640–3. 32171506 10.1016/j.jogc.2019.11.072

[53] Dayan N, Shapiro GD, Luo J, Guan J, Fell DB, Laskin CA, Basso O, Park AL, Ray JG. Development and internal validation of a model predicting severe maternal morbidity using pre-conception and early pregnancy variables: a population-based study in Ontario, Canada. BMC Pregnancy Childbirth. 2021;21:679. 10.1186/s12884-021-04132-6. 34615477 10.1186/s12884-021-04132-6 PMC8496026

[54] Leonard SA, Kennedy CJ, Carmichael SL, Lyell DJ, Main EK. An expanded obstetric comorbidity scoring system for predicting severe maternal morbidity. Obstet Gynecol. 2020;136:440–9. 10.1097/AOG.0000000000004022. 32769656 10.1097/AOG.0000000000004022 PMC7523732

[55] Rial-Crestelo M, Lubusky M, Parra-Cordero M, et al. Term planned delivery based on fetal growth assessment with or without the cerebroplacental ratio in low-risk pregnancies (RATIO37): an international, multicentre, open-label, randomised controlled trial. Lancet. 2024;403:545–53. 10.1016/S0140-6736(23)02228-6. 38219773 10.1016/S0140-6736(23)02228-6

[56] Sovio U, White IR, Dacey A, Pasupathy D, Smith GCS. Screening for fetal growth restriction with universal third trimester ultrasonography in nulliparous women in the pregnancy outcome prediction (POP) study: a prospective cohort study. Lancet. 2015;386:2089–97. 10.1016/S0140-6736(15)00131-2. 26360240 10.1016/S0140-6736(15)00131-2 PMC4655320

[57] Ferreira I, Simões J, Pereira B, et al. Ensemble learning for fetal ultrasound and maternal-fetal data to predict mode of delivery after labor induction. Sci Rep. 2024;14:15275. 10.1038/s41598-024-65394-6. 38961231 10.1038/s41598-024-65394-6 PMC11222528

