# Supplementary material for: Does infant birthweight percentile identify mothers at risk of severe morbidity? A Canadian population-based cohort study
Source: Matern Health Neonatol Perinatol. 2025 Jul 3;11:19. doi: 10.1186/s40748-025-00217-8 (PMC12225049; doi:10.1186/s40748-025-00217-8)
Supplement: Supplementary file 1 — Supplementary Material 1 [file 40748_2025_217_MOESM1_ESM.docx]

**eTable 1. Variables used to define cohort entry and exclusion criteria, study exposures, outcomes and adjustment variables.**

| **Assessment** | **Timing** | **Disease or procedure** | **CIHI-DAD ICD-9 [ICD-10-CA] and CCI (bolded) codes {or other source in parentheses}** | **OHIP ICD-9 codes** | **PubMed link to related validation studies for some codes** |
| --- | --- | --- | --- | --- | --- |
| ***Cohort entry criterion*** | September 1, 2002 to March 31, 2020 | All obstetrically delivered  mothers and infants in the province of Ontario at ≥ 23 weeks’ gestation | Main patient service code indicating  “obstetrical delivery” (MOMBABY - includes linked CIHI-DAD inpatient admission records of delivering mothers and their infants) | -- | -- |
| ***Cohort exclusion criteria*** | At the index delivery (maternal) or birth (infant) hospitalization | Invalid maternal healthcare number (HCN), sex, birth date, death date or discharge date | 1. Invalid HCN; or 2. {RPDB} Birth Date is missing or Sex is missing or male; or 3. Admission Date follows the Discharge Date at the index delivery or the {RPDB} Death Date | -- | -- |
|  | Same as above | Mother is not an Ontario resident | {RPDB} Province Number is not ‘35’ | -- | -- |
|  | Same as above | Maternal age < 10 or > 55 years | {RPDB} Age | -- | -- |
|  | Same as above | Stillbirth or intrauterine fetal death | On the infant birth record: [P95]; or  On the maternal delivery record: [Z37.1, Z37.3, Z37.4, Z37.6, Z37.7, O36.4] | -- | -- |
|  | Same as above | Multiple birth | On the infant birth record: [Z38.3-Z38.6, Q89.4]; or  On the maternal delivery record: [Z37.2-Z37.7, Z37.90, O30, O31] | -- | -- |
|  | Same as above | Gestational age at birth is missing, < 23 weeks or > 42 weeks | Clinical Gestation Weeks at Delivery | -- | -- |
|  | Same as above | Invalid liveborn infant HCN, sex, birth date, death date or discharge date | Discharge Disposition is alive and   1. Invalid HCN; or 2. Sex or Birth Date is missing {RPDB}; or 3. Sex or Birth Date differs between CIHI-DAD and {RPDB}; or 4. Death Date precedes the Birth Date {RPDB} | -- | -- |
|  | Same as above | Liveborn birthweight is missing or implausible for gestational age | Newborn Weight, Clinical Gestation Weeks at Delivery, and Sex | -- | -- |
| ***Primary outcome*** | From ≥ 23 weeks’ gestation up to ≤ 42 days after the index birth date | Any Severe Maternal Morbidity (SMM) or mortality in the mother (0, 1 or more) | Severe preeclampsia and hemolysis, elevated liver enzymes and low platelets (HELLP) syndrome: [O14.1, O14.2]  Eclampsia: [O15]  Cerebral venous thrombosis in pregnancy, or the puerperium: [O22.5, O87.3]  Acute fatty liver with red blood cell (RBC) transfusion or plasma transfusion:  [O26.6] + (CIHI-DAD BTREDBC=1 or CIHI-DAD BTPLASMA=1)  Pulmonary, cardiac, and CNS complications of anaesthesia during pregnancy, the puerperium, or labour and delivery: [O29.0, O29.1, O29.2, O89.0, O89.1, O89.2, O74.0, O74.1, O74.2, O74.3]  Placenta previa with hemorrhage with RBC transfusion: [O44.1] + CIHI-DAD BTREDBC=1  Placental abruption with coagulation defect: [O45.0]  Antepartum hemorrhage with coagulation defect: [O46.0]  Intrapartum hemorrhage with coagulation defect: [O67.0]  Intrapartum hemorrhage with RBC transfusion: [O67] + CIHI-DAD BTREDBC=1  Rupture of the uterus with RBC  transfusion, procedures to the uterus or hysterectomy: [O71.0 or O71.1] + any of the following:   - CIHI-DAD BTREDBC=1, or - (**1.RM.13, 1.KT.51, 5.PC.91.LA** or **5.PC.91.HV**) + CIHI-DAD BTREDBC=1, or - (**5.MD.60.RC, 5.MD.60.RD, 5.MD.60.KE, 5.MD.60.CB or 1.RM.89.LA**^c^)**,** or - **1.RM.87.LA-GX**   ^c^ NOTE: **1.RM.89.LA** is included only if codes **1.PL.74, 1.RS.74 or 1.RS.80** are NOT also present  Postpartum hemorrhage with RBC transfusion, procedures to the uterus or hysterectomy: [O72] + any of the following:   - CIHI-DAD BTREDBC=1, or - (**1.RM.13, 1.KT.51, 5.PC.91.LA or 5.PC.91.HV**) + DAD BTREDBC=1, or - (**5.MD.60.RC, 5.MD.60.RD, 5.MD.60.KE, 5.MD.60.CB or 1.RM.89.LA**^d^), or - **1.RM.87.LA-GX**   ^d^ NOTE: **1.RM.89.LA** is included only if  codes **1.PL.74, 1.RS.74 or 1.RS.80** are  NOT also present  Cardiac conditions: [O74.2, O89.1, O90.3, I21, I22, I42, I43, I46, I49.0, I50, J81], **1.HZ.09, 1.HZ.30**  Obstetric shock: [O75.1, R57, T80.5, T88.6]    Septicemia during labour: [O75.3]  Complications of obstetric surgery and procedures: [O75.4]  Puerperal sepsis: [O85]  Obstetric embolism: [O88]  Acute renal failure: [O90.4, N17, N19, N99.0]  Disseminated intravascular coagulation:  [D65]  Sickle cell anemia with crisis: [D57.0]  Acute psychosis: [F53.1, F23]  Status epilepticus: [G41]  Cerebral edema or coma: [G93.6, R40.2]  Cerebrovascular diseases: subarachnoid and intracranial hemorrhage, cerebral infarction, stroke: [I60, I61, I62, I63, I64]  Status asthmaticus: [J45.01, J45.11, J45.81, J45.91]  Adult respiratory distress syndrome: [J80]  Acute abdomen: [K35, K37, K65, N73.3, N73.5]  Hepatic failure: [K71, K72]  Assisted ventilation through endotracheal tube: **1.GZ.31.CA-ND**  Assisted ventilation through tracheostomy: **1.GZ.31.CR-ND**  Hysterectomy: **5.MD.60.RC, 5.MD.60.RD, 5.MD.60.KE, 5.MD.60.CB, 1.RM.89.LA** (exclude if **1.PL.74, 1.RS.74 or 1.RS.80** code also present) **or 1.RM.87.LA-GX**  Dialysis: **1.PZ.21**  Evacuation of incisional hematoma with RBC transfusion: **5.PC.73.JS** + CIHI-DAD BTREDBC=1  Repair of bladder, urethra, or intestine: **5.PC.80.JR, 1.NK.80, 1.NM.80**  Procedures to the uterus/pelvic vessels with RBC transfusion: (**1.RM.13, 1.KT.51, 5.PC.91.LA or 5.PC.91.HV**) + CIHI-DAD BTREDBC=1  Surgical or manual correction of inverted uterus for vaginal births only: **5.PC.91.HQ or 5.PC.91.HP**, restricted to vaginal births (i.e., absence of caesarean **5.MD.60**)  Reclosure of caesarean wound: (**5.PC.80.JM or 5.PC.80.JH**) + CIHI-DAD BTREDBC=1  Curettage with RBC transfusion: (**5.PC.91.GA, 5.PC.91.GC or 5.PC.91.GD**) + CIHI-DAD BTREDBC=1  Maternal ICU admission: CIHI-DAD Special Care Unit 10, 20, 25, 30, 35, 40, 45, 60, 80 | -- | Previous Canadian SMM definition by the Canadian Perinatal Surveillance System (See https://www.ncbi.nlm.nih.gov/pmc/articles/PMC1216316/) |
| ***Secondary outcome*** | From the index birth date up to ≤ 42 days after the index birth date | Any SMM or mortality in the mother (0, 1 or more) | Same as above | -- | Same as above |
| ***Main study exposure*** | At the index live birth hospitalization | Birthweight for gestational age percentile | Newborn Weight, Clinical Gestation Weeks at Delivery, and Sex  {General population birthweight percentiles were based on Ontario Vital Statistics Births 2002-2011} | -- | <https://www.ncbi.nlm.nih.gov/pubmed/29117948> |
| ***Secondary exposure*** | Same as above | Birthweight for gestational age percentile by maternal world region of origin (Canada/Long-term resident, Caribbean/Sub-Saharan Africa, East Asia/Pacific, Hispanic America, Middle East/North Africa, South Asia, Western Nations/Europe, Unknown) | Newborn Weight, Clinical Gestation Weeks at Delivery, and Sex  {Maternal ethnicity-specific birthweight percentiles were based on Ontario Vital Statistics Births 2002-2011}  {Maternal World Region of Origin from IRCC Permanent Resident Database} | -- |  |
| ***Stratification variables*** | ≤ 24 months before the index delivery date | Pre-pregnancy diabetes mellitus | 250, 648.8, [E10, E11, E13, E14, O24.5, O24.6, O24.7] | 250 | <https://www.ncbi.nlm.nih.gov/pubmed/11874939> |
|  | Same as above | Pre-pregnancy chronic hypertension | 401, 405, 642.0-642.2, 642.7 [I10, I15, O10, O11] | 401 | <https://www.ncbi.nlm.nih.gov/pubmed/19858407> |
|  | From the index pregnancy prenatal record | Pre-pregnancy Body Mass Index (≥ 30 or < 30 kg/m^2^) | BMI {BORN} ^a^ | -- |  |
|  | At the index birth hospitalization | Parity (parous or nulliparous) | CIHI-DAD Number of previous live births | -- |  |
|  | Same as above | Mode of birth (vaginal or Caesarean section) | **5.MD.60** | -- |  |
| **Covariates** | At the time of the index delivery hospitalization | Maternal age (continuous) | CIHI-DAD Age | -- |  |
|  | Same as above | Residential Income Quintile (Q) (1/missing, 2, 3, 4, 5) | {Statistics Canada census data} | -- |  |
|  | Same as above | Rural residence (rural/missing, urban) | {Statistics Canada census data} | -- |  |
|  | ≤ 24 months before the index delivery date | Pre-pregnancy diabetes mellitus | 250, 648.8, [E10, E11, E13, E14, O24.5, O24.6, O24.7] | 250 | <https://www.ncbi.nlm.nih.gov/pubmed/11874939> |
|  | Same as above | Pre-pregnancy chronic hypertension | 401, 405, 642.0-642.2, 642.7 [I10, I15, O10, O11] | 401 | <https://www.ncbi.nlm.nih.gov/pubmed/19858407> |
| ***Other variables*** | At the index birth hospitalization | Infant sex (male or female) | CIHI-DAD Sex | -- |  |
|  | From the index birth hospitalization up to 365 days thereafter | Congenital or chromosomal anomaly diagnosed in the infant’s 1^st^ year of life | Diagnosis code starting with ‘Q’ | -- |  |

^a^ Limited to births from April 2006 to March 2014.

BORN: Better Outcomes Registry & Network; CCI: Canadian Classification of Health Interventions; CIHI: Canadian Institutes of Health Information; DAD: Discharge Abstract Database; ICD-9: International Classification of Diseases, 9th Revision; ICD-10-CA: International Classification of Diseases and Health Related Problems, 10th Revision, Canada; IRCC: Immigration, Refugees and Citizenship Canada; OHIP: Ontario Health Insurance Plan; RPDB: Registered Persons Database (contains demographic information and encrypted healthcare numbers for all individuals eligible for OHIP)

**Table S2.** **Plausible relation between poor fetal growth and the various indicators of severe maternal morbidity (SMM) (for *Additional analysis B)*.** SMM indicators were separated, *a priori*, into those that are **more likely** or **less likely** related to poor fetal growth. Specific ICD-10-CA or CCI codes are identified.

|  | **SMM Indicator** | **ICD-10-CA or CCI Codes** |
| --- | --- | --- |
| **SMM indicators more likely to be related to poor fetal growth** | Severe pre-eclampsia, HELLP syndrome | O14.1, or O14.2 |
|  | Eclampsia | O15 |
|  | Placental abruption with coagulation defect | O45.0 |
|  | Antepartum hemorrhage with coagulation defect | O46.0 |
|  | Maternal ICU admission | SCU in (‘10’, ’20’, ’25’, ’30’, ’35’, ’40’,’45’,’60’,’80’) |
|  | Cardiomyopathy, cardiac arrest and resuscitation, myocardial infarction, pulmonary edema and heart failure | O74.2, O89.1, O90.3, I21, I22, I42, I43, I46, I49.0, I50, J81, 1.HZ.09 or 1.HZ.30 |
|  | Acute renal failure | O90.4, N17, N19 or N99.0 |
|  | Dialysis | 1.PZ.21 |
|  |  |  |
| **SMM indicators less likely to be related to poor fetal growth** | Placenta previa with hemorrhage and red cell transfusion | O44.1 + CIHI BTREDBC = 1 |
|  | Intrapartum hemorrhage with coagulation defect | O67.0 |
|  | Intrapartum hemorrhage with red cell transfusion | O67 + CIHI BTREDBC = 1 |
|  | Postpartum hemorrhage with red cell transfusion, procedures to the uterus or hysterectomy | O72 + any of the following: BTREDBC = 1, or (1.RM.13, 1.KT.51, 5.PC.91.LA or 5.PC.91.HV) + BTREDBC = 1, or (5.MD.60.RC, 5.MD.60.RD, 5.MD.60.KE, 5.MD.60.CB or 1.RM.89.LA), or 1.RM.87.LA-GX. **NOTE**: **1.RM.89.LA** is included only if codes 1.PL.74, 1.RS.74 or 1.RS.80 are NOT also present. |
|  | Curettage with red cell transfusion | (5.PC.91.GA, 5.PC.91.GC, 5.PC.91.GD) + CIHI BTREDBC = 1 |
|  | Complications of obstetric surgery and procedures | O75.4 |
|  | Evacuation of incisional hematoma with RBC transfusion | 5.PC.73.JS + CIHI BTREDBC = 1 |
|  | Repair of bladder, urethra, or intestine | 5.PC.80.JR, 1.NK.80, 1.NM.80 |
|  | Reclosure of caesarean wound with RBC transfusion | (5.PC.80.JM, 5.PC.80.JH) + CIHI BTREDBC = 1 |
|  | Procedures to the uterus with RBC transfusion | (1.RM.13, 1.KT.51, 5.PC.91.LA, 5.PC.91.HV) + CIHI BTREDBC = 1 |
|  | Caesarean hysterectomy, hysterectomy using an open approach | 5.MD.60.RC, 5.MD.60.RD, 5.MD.60.KE, 5.MD.60.CB, 1.RM.89.LA (exclude if 1.PL.74, 1.RS.74 or 1.RS.80 code also present), 1.RM.87.LA-GX |
|  | Puerperal sepsis | O85 |
|  | Septicemia during labour | O75.3 |
|  | Obstetric embolism | O88 |
|  | Obstetric shock | O75.1, R57, T80.5 or T88.6 |
|  | Disseminated intravascular coagulation | D65 |
|  | Assisted ventilation through endotracheal tube | 1.GZ.31.CA-ND |
|  | Assisted ventilation through tracheostomy | 1.GZ.31.CR-ND |
|  | Rupture of the uterus with red cell transfusion, procedures to the uterus or hysterectomy | (O71.0 or O71.1) + any of the following: CIHI BTREDBC = 1, or (1.RM.13, 1.KT.51, 5.PC.91.LA or 5.PC.91.HV) + CIHI BTREDBC = 1, or (5.MD.60.RC, 5.MD.60.RD, 5.MD.60.KE, 5.MD.60.CB or 1.RM.89.LA**^a^**), or 1.RM.87.LA-GX. **NOTE**: 1.RM.89.LA is included only if codes 1.PL.74, 1.RS.74 or 1.RS.80 are NOT also present |
|  | Cerebral venous thrombosis in pregnancy | O22.5 |
|  | Cerebral venous thrombosis in the puerperium | O87.3 |
|  | Subarachnoid and intracranial hemorrhage, cerebral infarction | I60, I61, I62, I63, or I64 |
|  | Acute fatty liver with red cell transfusion or plasma transfusion | O26.6 + (CIHI BTREDBC = 1 or CIHI BTPLASMA = 1) |
|  | Cerebral edema or coma | G93.6 or R40.2 |
|  | Pulmonary, cardiac, and CNS complications of anesthesia during pregnancy, labour, delivery or the puerperium | O29.0, O29.1, O29.2, O89.0, O89.1, O89.2, O74.0, O74.1, O74.2 or O74.3 |
|  | Status asthmaticus | J45.01, J45.11, J45.81 or J45.91 |
|  | Adult respiratory distress syndrome | J80 |
|  | Hepatic failure | K71 or K72 |
|  | Acute abdomen | K35, K37, K65, N73.3 or N73.5 |
|  | Surgical or manual correction of inverted uterus for vaginal births only | 5.PC.91.HQ or 5.PC.91.HP, restricted to vaginal births (i.e., absence of caesarean 5.MD.60) |
|  | Sickle-cell anemia with crisis | D57.0 |
|  | Acute psychosis | F53.1 or F23 |
|  | Status epilepticus | G41 |

ICD-10-CA: International Classification of Diseases and Health Related Problems, 10th Revision, Canada; CCI: Canadian Classification of Health Interventions.

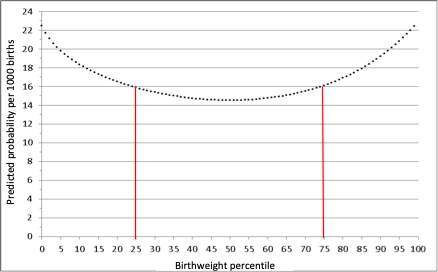
**Figure S2. Predicted probability of severe maternal morbidity or maternal death (SMMM) from 23 weeks' gestation up to 42 days postpartum, in relation to newborn weight percentile.** The predicted probability (curved dashed line) was calculated using univariable fractional polynomial regression. The vertical red lines cross at the 25^th^ and 75^th^ percentiles of newborn weight.

**Figure S3 *(Additional analysis A*). Risk of severe maternal morbidity or death from 23 weeks' gestation up to 42 days postpartum, in relation to newborn weight percentile, based on ethnicity-specific birthweight curves**. Relative risks (RR) are adjusted for maternal age, income quintile aand rural residence – each at the time of the index birth -- as well as diabetes mellitus and chronic hypertension within 2 years before the index birth.

**Figure S4. Risk of severe maternal morbidity or death from 23 weeks' gestation up to 42 days postpartum, in relation to newborn weight percentile, stratified by pre-pregnancy body mass index (BMI).** Relative risks (RR) are adjusted for maternal age, income quintile and rural residence – each at the time of the index birth -- as well as diabetes mellitus and chronic hypertension within 2 years before the index birth. This analysis is limited to 308,023 pregnancies with known maternal pre-pregnancy BMI.

**Figure S5. Risk of severe maternal morbidity or death from 23 weeks' gestation up to 42 days postpartum, in relation to newborn weight percentile, stratified by parity.** Relative risks (RR) are adjusted for maternal age, income quintile and rural residence – each at the time of the index birth -- as well as diabetes mellitus and chronic hypertension within 2 years before the index birth.

**Figure S6. Risk of severe maternal morbidity or death from 23 weeks' gestation up to 42 days postpartum, in relation to newborn weight percentile, stratified by mode of delivery.** Relative risks (RR) are adjusted for maternal age, income quintile and rural residence – each at the time of the index birth -- as well as diabetes mellitus and chronic hypertension within 2 years before the index birth.

**Figure S7 (*Additional analysis B*). Risk of severe maternal morbidity (SMM) from 23 weeks' gestation up to 42 days postpartum, in relation to newborn weight percentile,** separated into SMM indicators **more likely** or **less likely** related to poor fetal growth (see Table S2). Relative risks (RR) are adjusted for maternal age, income quintile and rural residence – each at the time of the index birth -- as well as diabetes mellitus and chronic hypertension within 2 years before the index birth.
